# Experience With Palbociclib in Metastatic Breast Cancer Patients Managed Under a Government Health Scheme at a Cancer Care Center in Southern India

**DOI:** 10.7759/cureus.70394

**Published:** 2024-09-28

**Authors:** Priyadharshini Eashwar, Deepak C Yadlapalli, Muralidhar Gullipalli

**Affiliations:** 1 Medical Oncology, GSL (Ganni Subbalakshmi) Medical College, Rajamahendravaram, IND

**Keywords:** breast cancer, cdk 4/6 inhibitors, estrogen receptor, hormone receptor-positive, palbociclib, progesterone receptor

## Abstract

Introduction

According to Globocan 2022, breast cancer ranks number one among the cancers worldwide. South Asian women have a higher incidence compared to Westerners. Estrogen receptor (ER) and progesterone receptor (PR) positive tumors, termed hormonal receptor-positive tumors, account for most breast cancer presentations. In India, advanced-stage presentations are more common. In metastatic hormone receptor-positive breast cancer, hormonal therapy combined with cyclin-dependent kinase (CDK) 4/6 inhibitors is the standard treatment.

Aim

This study aimed to analyze the experience with generic palbociclib provided under the Government Health Scheme for metastatic hormone receptor-positive breast cancer at our institution.

Methods

This retrospective study was conducted on breast cancer patients admitted to a tertiary care center in South India. The data of ER and PR receptor-positive metastatic breast cancer patients who received palbociclib were identified and reviewed using medical records from 2023 to 2024.

Results

A total of 238 patients were analyzed, of which 41 received palbociclib for hormone receptor-positive metastatic breast cancer. The median age was 49 (33-75), with 53.5% (22) of women above 50. Denovo stage IV presentation was observed in 21 patients (51.2%), while progression to stage IV disease was noted in 11 patients (26.8%), and stages II and III were noted in nine patients (22%). Invasive ductal carcinoma was the most common histology. All patients were ER-positive, and 38 (92.7%) were PR-positive. About 17 patients (41.5%) had visceral metastasis, and 12 (29.3%) had bone-only metastasis. Local recurrence was seen in six patients (14.6%), and bone with visceral metastasis was seen in another six patients (14.6%). Progression within one year of hormonal therapy initiation was observed in 50% (10) of patients. Among 21 patients with upfront metastasis, nine were treated with prior chemotherapy. All patients were given 125 mg of oral palbociclib. Fatigue was the most common side effect in 34.1% (14), followed by myalgia in 21.9% (9), low hemoglobin levels of less than 8 g/dl in 14.6% (6), and nausea and vomiting in only 9.8% (4) of patients.

Conclusion

Hormone therapy combined with CDK4/6 inhibitors is the backbone of treatment for metastatic hormone-positive breast cancer. However, in developing countries like India, where most patients come from rural areas, using innovator palbociclib may not be feasible for many. With the availability of generic palbociclib under the Government Health Scheme, patients of metastatic hormone receptor-positive breast cancer will receive the protocol-based treatment.

## Introduction

Breast cancer represents the most prevalent malignancy affecting women globally, including in India. The Globocan 2022 data reveals that breast cancer represents 26.6% of the total cancer cases among females in India. In 2022, the incidence of breast cancer among women reached 2.3 million cases, resulting in approximately 670,000 mortalities worldwide. In India, breast cancer represents the most significant proportion of cancer cases, with 192,020 new diagnoses recorded. This accounts for 13.6% of the total cancer patient population, with a prevalence exceeding 26% among women and a mortality of 98, 33 cases [1]. Breast cancer incidence is higher in women of South Asian origin when compared to their Western counterparts [2]. This is mainly attributed to the lack of awareness, screening, socioeconomic status, and diversity in healthcare affordability. More than 50% of patients in India with carcinoma breast present with advanced stages III and IV disease [3]. The higher the stage, the greater the mortality. The five-year overall survival rates of stages I, II, III, and IV are 95%, 92%, 70%, and 21%, respectively [4].

Breast carcinoma is a heterogeneous malignancy. Breast cancer is classified into four molecular subtypes based on positivity for estrogen receptor (ER), progesterone receptor (PR), Her2 neu, and Ki67 index: luminal A, luminal B, Her 2-enriched, and triple-negative [5]. The incidence of hormone-positive breast cancers ranges from 45% to 50% [6], with around 60% of these patients in stage IV [7].

Patients with hormone-positive breast cancer can present with three phenotypes: ER+/PR+, ER+/PR-, and ER-/PR+. The clinical features of ER+/PR+ and ER+/PR- tumors are seen in older females, well-differentiated, and early-stage diseases compared to ER-/PR+ tumors, which predominantly affect younger patients, are advanced, and poorly differentiated [8]. In terms of survival, double hormone-positive patients had better survival compared to single hormone-positive patients [9].

It is proven beyond doubt that adjuvant hormonal therapy achieves better disease-free survival, recurrence-free survival, overall survival, and reduced contralateral breast malignancy in non-metastatic hormone-positive breast cancer [10,11].

Metastatic hormone-positive patients are categorized as having endocrine-sensitive or endocrine-resistant HR-positive disease [12]. Endocrine-sensitive patients have de novo metastatic disease or recurrence more than a year after completing adjuvant endocrine treatment. In contrast, endocrine-resistant patients experience recurrence while receiving adjuvant endocrine treatment or within one year of completing it or have progressed while receiving endocrine therapy for advanced disease [13].

The approval of cyclin D kinase 4/6 (CDK 4/6) inhibitors in 2015 was a game changer in the treatment of metastatic hormone-positive carcinoma breast. Palbociclib, ribociclib, and abemaciclib are approved for treating metastatic endocrine breast cancer patients along with letrozole or fulvestrant. CDK on phosphorylation of retinoblastoma (RB) tumor suppressor protein leads to activation of E2F family transcription factors. This in turn leads to DNA replication and cell cycle progression. CDK4/6 inhibitors hinder the phosphorylation and inhibit the transcription activation necessary for cell proliferation [14].

Palbociclib (IBRANCE) was the first in CDK4/6 inhibitors to receive Food and Drug Administration (FDA) approval in February 2015 for use in postmenopausal women with advanced breast cancer. The history of palbociclib dates as early as the 1970s, with the discovery of CDKs being an important cell cycle regulator by Sir Paul Nurse, Tim Hunt, and Leland Hartwell, winning them the Nobel prize. Parke-Davis and Co. first developed the drug in 2000, which later was taken over by Pfizer in 2003. Initially developed under PD-0332991, the pre-clinical trial showed positive results in the breast cancer cell line, showing ER and PR positivity; this led to further investigation of the drug in the PALOMA 1 trial [15].

In the open-label phase two PALOMA 1 trial, patients with hormone-positive breast cancer on palbociclib with letrozole showed better progression-free survival when compared to letrozole alone. These results lead to early FDA approval and further evaluation of efficacy and safety with confirmation of the results in PALOMA 2 [16]. The major side effects noted were neutropenia, leukopenia fatigue, arthralgia, nausea, and alopecia.

IBRANCE, the original molecule by Pfizer, costs around 70,000 Indian rupees (125 mg 21 tablets), which made it difficult for many Indian patients to afford. The development of Indian generic palbociclib, 125 mg 21 tablets for 7000 Indian rupees, paved the path for Indian patients. However, while Pfizer's patent for palbociclib was extended until 2027 in the United States, the Indian Law did not apply for an extension, which led many other Indian pharmaceutical companies to produce generic palbociclib. Before the accessibility of palbociclib in the Indian market, patients with metastatic hormone-positive breasts were either treated with systemic chemotherapy or alternative hormone therapy.

As there are very few reports on generic palbociclib, this study analyzed the real-world scenario of using generic palbociclib in our daily practice. In our institution, the majority of our patients come from rural areas and benefit from the drug provided through the Government Health Scheme.

## Materials and methods

A retrospective observational single-institution study was conducted at a tertiary care center in South India from April 2023 to March 2024. Data were collected from the medical records of patients who received palbociclib during this period.

The study was performed at Ganni Subbulakshmi Garu Cancer Hospital and Research Center, Rajamahendravaram, Andhra Pradesh, India, and ethical approval was obtained (GSLMC/RC:1248A-EC/1248A-03/2024). The medical records of all patients treated at our center between January 2023 and December 2023 were obtained and subjected to further analysis. All patients received accurate therapy at our institute and were uniformly managed by a dedicated team.

The study included 238 patients, and the majority were from rural areas. All patients received generic palbociclib under the Government Health Scheme from 2023. Patients above 18 years old, with an Eastern Cooperative Oncology Group (ECOG) performance status of 0 to 2, and hormone-positive carcinoma breast, presenting as either de novo or disease progression to a distant site where a local form of therapy was not feasible, were included in the study. Patients with an ECOG score of 3 or greater and comorbidities like abnormal renal and liver function were excluded.

The diagnosis of breast cancer was predominantly established through clinical presentation, imaging techniques including mammography, ultrasound, or magnetic resonance imaging (MRI), as well as histopathological methods. Staging for localized tumors was done using chest X-rays and ultrasound, while for advanced and metastatic tumors, staging was conducted using CT scans, bone scans, and positron emission tomography (PET). The classification of tumors based on ER, PR, and human epidermal growth factor receptor 2 (HER2) status was done through immunohistochemistry (IHC) analysis (PathnSitu Biotechnologies, Hyderabad, Telangana, India). The classification of hormone receptor-positive cases was established based on the expression levels of ER, PR, and HER2, assessed through IHC or fluorescence in situ hybridization (FISH) amplification positivity.

Patient demographics and clinical characteristics were recorded, along with tumor stage, grading, and histology. Tumor staging and grading followed the guidelines of the American Joint Committee on Cancer (AJCC) eighth edition, and the Nottingham histologic score system was used for the tumor histological grading, which evaluates the parameters such as tubule formation, nuclear pleomorphism, and mitotic activity. According to the staging criteria, patients were classified into three primary categories: early-stage breast cancer (EBC), which includes T1-2, N0-1, and M0 stages; locally advanced breast cancer (LABC), characterized by T-stage ≥ T3 and/or N-stage ≥ N2, M0; and metastatic breast cancer (MBC), defined by the presence of distant metastasis.

All patients received oral palbociclib tablet 125 mg for 21 days with a seven-day break, along with oral letrozole tablet 2.5 mg or intramuscular injection of fulvestrant on days 1 and 14 in a 28-day cycle, followed by once every 28 days subsequently. Premenopausal or perimenopausal patients were given luteinizing hormone-releasing hormone (LHRH) as per current guidelines.

Patients were advised to take palbociclib with food, and in cases of vomiting or missed doses, they were instructed not to take an extra dose on the same day but to continue the tablet at the usual time the following day. Patients were encouraged to take the medication regularly, at the same time every day, and to swallow it whole without breaking the tablet.

Patients who were HER2-positive (triple positive: ER, PR, and HER2) were given the anti-HER2 monoclonal antibody, trastuzumab injection intravenously every three weeks, with an initial dose of 8 mg/kg in the first cycle followed by 6 mg/kg in subsequent cycles. Patients with bone metastases received bisphosphonates monthly.

Complete blood count, serum creatinine, serum total bilirubin, ECG, and 2D-ECHO were conducted before starting palbociclib as part of the institutional protocol. Hematological evaluations were done on day 15 for the first two cycles, followed by every four weeks. Patients were assessed for symptoms of drug toxicities every month, and imaging evaluations were performed only when patients presented with new symptoms due to financial constraints. Adverse effects noted in the medical records were taken for analysis. Statistical analysis was performed using SPSS software version 20.0 (IBM Corp., Armonk, NY) and MS Excel 2016 (Microsoft Corp., Redmond, WA).

## Results

During the period from January 2023 to December 2023, a cohort of 238 patients diagnosed with breast carcinoma was documented at our institution, of which 102 (43%) were hormone-positive. Of these, 41 patients with metastatic hormone-positive breast cancer were on generic palbociclib. The median age was 49 years (range: 33-75). Among the 41 patients, 53.7% (n = 22) were over 50 years of age at the time of diagnosis. De novo metastasis was observed in 21 patients (51.2%), while the remaining patients had progressed from stage 2 (26.8%, n = 11) and stage 3 (22%, n = 9), respectively. Intraductal carcinoma was the most common histology, accounting for 97.5% (n = 40).

All patients were positive for ER, 92.7% (n = 38) were positive for PR, and 24.3% (n = 10) were triple positive for ER, PR, and HER2. Around 93% (n = 38) were ER+/PR+, 7.3% (n = 3) were ER+/PR-, and 2.4% (n = 1) were ER+/PR-/HER2+. Triple positivity was found in 24.3% (n = 10) of patients.

The most common site of metastasis was visceral organs (liver or lung), accounting for 41.5% (n = 17), followed by bone-only metastasis at 29.3% (n = 12). Recurrence at the chest wall was observed in 14.3% (n = 6), and 14.6% (n = 6) had both visceral and bone metastasis. Detailed patient characteristics are presented in Table 1.

**Table 1:** Patient and tumor characters (N = 41) IDC: Invasive lobular carcinoma; ILC: Invasive ductal carcinoma; IHC: Immunohistochemistry; ER: Estrogen receptor; PR: Progesterone receptor; HER2: Human epidermal growth factor receptor 2; ECOG: Eastern Cooperative Oncology Group.

		Number (%)
Age	Median age	Years
	<50 years	19 (46.3%)
	≥50 years	22 (53.7%)
Stage	Stage II	11 (26.8%)
	Stage III	9 (22%)
	Stage IV	21 (51.2%)
Histology	IDC	40 (97.5%)
	ILC	1 (2.5%)
IHC	ER	41(100%)
	PR	38 (92.7%)
	ER/PR and HER2	10 (24.3%)
ECOG	0	10 (24.3%)
	1	24 (58.5%)
	2	7 (17.2%)
Metastasis	Visceral	17 (41.5%)
	Bone only	12 (29.3%)
	Local recurrence	6 (14.6%)
	Visceral + bone	6 (14.6%)

Among patients with non-metastatic disease at the time of diagnosis, who had completed surgery, adjuvant chemotherapy, and radiation, 55% (n = 11) had progressed on letrozole and 45% (n = 9) on tamoxifen. Fifty percent (n = 10) of these patients progressed within one year, 40% (n = 8) progressed within five years, and 10% (n = 2) progressed after more than five years (Table 2).

**Table 2:** Non-metastatic patient at the time of diagnosis (N = 20)

		N (%)
Progression in hormonal therapy (N = 20)	Letrozole	11 (55%)
	Tamoxifen	9 (45%)
Time of progression (N = 20)	Within 1 year	10 (50%)
	1 to 5 years	8 (40%)
	>5 years	2 (10%)

Of the 21 patients who presented with metastasis at the time of diagnosis, nine patients had prior chemotherapy before starting palbociclib; 77.8% had at least one previous chemotherapy; 22.2% had two lines of prior chemotherapy, and none had more than two prior chemotherapy (Table 3).

**Table 3:** Upfront metastasis at the time of diagnosis (N = 21)

		N (%)
Prior chemotherapy	Not received	12 (57.1%)
	Received	9 (42.9%)
Number of previous chemotherapy (N = 9)	1 regimen	7 (77.8%)
	2 regimens	2 (22.2%)
	More than 2	0

Thirty-one patients were started on palbociclib and letrozole, while the remaining patients received fulvestrant. Approximately 78% (n = 32) of patients took palbociclib for up to six months, and 22% (n = 9) took palbociclib for more than six months. Nausea and vomiting were noted in 9.8% (n = 4) of patients, hemoglobin levels below 8 g/dl were seen in 17.1% (n = 7), 34% (n = 14) experienced fatigue, and 21.9% (n = 9) had myalgia. None of the patients experienced deep venous thrombosis, alopecia, or skin rashes (Table 4). Five patients enrolled in the study died.

**Table 4:** Adverse effects of palbociclib documented (N = 34) DVT: Deep vein thrombosis.

Adverse effects	%	N
Fatigue	41.2%	14
Myalgia	26.5%	9
Low Hb	20.6%	7
Nausea and vomiting	11.8%	4
DVT	-	-
Skin rash	-	-
Alopecia	-	-

## Discussion

Hormone-positive breast cancer is the most common subtype, and endocrine therapy has long been the backbone of treatment for metastatic hormone-positive breast cancer. Resistance to hormonal treatment is the prevalent reason for disease progression in patients with hormonal therapy. However, the approval of CDK4/6 inhibitors has shifted the treatment paradigm in recent years.

The overall incidence of hormone-positive breast cancer in India is approximately 56%, according to Pandit et al. [17]. There is scant data on palbociclib in the Indian setting, with financial constraints being the attributing factor, as most patients at our center are from rural areas [18]. Since 2023, our patients have received generic palbociclib under the Government Health Scheme.

As per Gogia et al., the prevalence of hormone-positive breast cancer in India is around 59% [3]. In our study, the median age was 49 years, which is younger compared to Indian data according to Agrawal et al. and Rath et al., where the median ages were 58 and 57 years, respectively [18,19].

Invasive ductal carcinoma was the predominant histology, similar to the global trends. In our study, 43% of patients were stage IV at the time of diagnosis, and 47% progressed from either early or locally advanced stages, which contrasts with Agrawal et al., where stage IV presentation was higher at 52% [18]. In our study, 17% of patients were triple positive, in contrast to other Indian studies that included only ER/PR-positive patients.

Visceral metastasis was the most common in our patients which is similar to Agrawal et al.; however, bone-only metastasis occurred in 32% of patients, which was higher than the 17% reported in other studies [18]. In contrast to Finn et al., our study had more patients with visceral metastasis than non-visceral metastasis [16]. It is important to note that bone-only metastasis in our study was higher compared to Pareek et al., where the incidence was found to be 25% [20].

Patients who were non-metastatic at diagnosis but progressed on hormonal therapy were within 12 months, which was similar when compared to other Indian and Western data.

Regarding therapy, all patients in the study received palbociclib. It was combined with either letrozole or fulvestrant. Anti-HER2 therapy was added for triple-positive patients, and zoledronic acid was administered to patients with bone metastasis.

Chemotherapy was administered to 75% of patients with de novo metastasis, in contrast to the PALOMA 2 trial, where patients received palbociclib upfront without prior chemotherapy [16]. In our study, 78% of patients received at least one chemotherapy regimen before palbociclib was given, and no one received more than two chemotherapy regimens. Patients received prior chemotherapy due to high disease burden or financial constraints in obtaining palbociclib before it was approved by the Government Health Scheme.

Palbociclib is a well-tolerated drug, with none of our patients experiencing severe side effects. Sixty-seven percent of our patients took palbociclib for up to six months. The most common side effects were fatigue (32%, n = 9) and myalgia (20%, n = 6). Diarrhea was noted in 7.1% (n = 2) of patients, which is consistent with the findings of Rath et al. [19]. Anemia was observed in 14.3% (n = 4), and no cases of neutropenia were reported. None of the patients experienced alopecia, rashes, or deep vein thrombosis. When comparing the adverse effects reported by Finn et al. [16], where neutropenia occurred in around 70% of cases in the Western data, our patients tolerated the drug well, as none experienced any neutropenia. Fatigue was the most common non-hematological side effect in our study.

Before the approval of CDK4/6 inhibitors, due to financial reasons, alternative treatment options were considered. At our institution, chemotherapy was provided to patients who were unable to receive palbociclib before the government scheme was initiated.

The limitations of our study include its retrospective nature, a smaller sample size, incomplete documentation of side effects, and response evaluation at a defined time. However, compliance with treatment was good, as most patients regularly reported to the hospital due to the government scheme. Despite these limitations, our results indicate that generic palbociclib has better compliance and manageable adverse effects in our patient population.

## Conclusions

Hormone therapy combined with CDK4/6 inhibitors is the backbone of treatment for metastatic hormone-positive breast cancer. However, in developing countries like India, where most patients come from rural areas, using innovator palbociclib may not be feasible for many. With the availability of generic palbociclib under the Government Health Scheme, patients can now receive the protocol-based treatment for their hormone receptor-positive MBC. Generic palbociclib is well tolerated, even in patients who had prior chemotherapy, and has led to better treatment adherence in our rural Indian population.
